# Microbiota changes in lactation in the short-beaked echidna (*Tachyglossus aculeatus*)

**DOI:** 10.1093/femsec/fiaf036

**Published:** 2025-04-07

**Authors:** Isabella Wilson, Tahlia Perry, Raphael Eisenhofer, Peggy Rismiller, Michelle Shaw, Frank Grutzner

**Affiliations:** School of Biological Sciences, The University of Adelaide, Adelaide 5005, Australia; School of Biological Sciences, The University of Adelaide, Adelaide 5005, Australia; Australian Research Council Centre of Excellence for Australian Biodiversity and Heritage, The University of Adelaide, Adelaide 5005, Australia; School of Biological Sciences, The University of Adelaide, Adelaide 5005, Australia; Centre for Evolutionary Hologenomics, Globe Institute, University of Copenhagen, Copenhagen 1353, Denmark; School of Biological Sciences, The University of Adelaide, Adelaide 5005, Australia; Pelican Lagoon Research and Wildlife Centre, Penneshaw 5222, Australia; School of Biological Sciences, The University of Adelaide, Adelaide 5005, Australia; Taronga Wildlife Nutrition Centre, Welfare, Conservation & Science, Taronga Conservation Society Australia, Mosman 2088, Australia; School of Biological Sciences, The University of Adelaide, Adelaide 5005, Australia

**Keywords:** Australia, metagenomics, milk, monotreme, pouch microbiota, wildlife conservation

## Abstract

Monotreme and marsupial development is characterized by a short gestation, with young exposed to the environment at an early developmental stage and supported by a long lactation in the pouch, pseudo-pouch, or burrow. The lack of a functional adaptive immune system in these altricial young raises questions about how they survive in a microbe-rich environment. Previous studies on marsupial pouches have revealed changes to pouch microbe composition during lactation, but no information is available in monotremes. We investigated changes in the echidna pseudo-pouch microbiota (*n* = 22) during different stages of the reproductive cycle and whether this differs between wild and zoo-managed animals. Metataxonomic profiling using 16S rRNA gene sequencing revealed that pseudo-pouch microbial communities undergo dramatic changes during lactation, with significant differences in taxonomic composition compared with samples taken outside of breeding season or during courtship and mating. This suggests that the echidna pseudo-pouch environment changes during lactation to accommodate young that lack a functional adaptive immune system. Furthermore, captivity was not found to have a significant effect on pseudo-pouch microbiota. This study pioneers pouch microbiota research in monotremes, provides new biological information on echidna reproduction, and may also provide information about the effects of captive management to inform breeding programmes in the future.

## Teaser

This first characterization of the pouch microbiota of an iconic Australian species, the short-beaked echidna, reveals extensive changes in lactation.

## Introduction

The reproductive microbiome, which includes vaginal, milk, and mammary microbiota, is increasingly being recognized for its contributions to infant health. The first major exposure to bacteria occurs during birth when the infant comes into contact with maternal vaginal, faecal, and skin microbiota (Palmer et al. 2007). This results in colonization of oral, skin, nasopharyngeal, and gastrointestinal habitats within the infant (Dominguez-Bello et al. 2010). Subsequent exposure through lactation, including microbes from the milk as well as the skin surrounding the nipple, is crucial for the colonization of the infant gut (Mueller et al. 2015, Keady et al. 2023). Milk provides antimicrobials that prevent the growth of pathogenic species, and in humans has been shown to contain prebiotics that support the growth of beneficial microbes (Barile and Rastall 2013, Gopalakrishna and Hand 2020).

**Figure 1.**
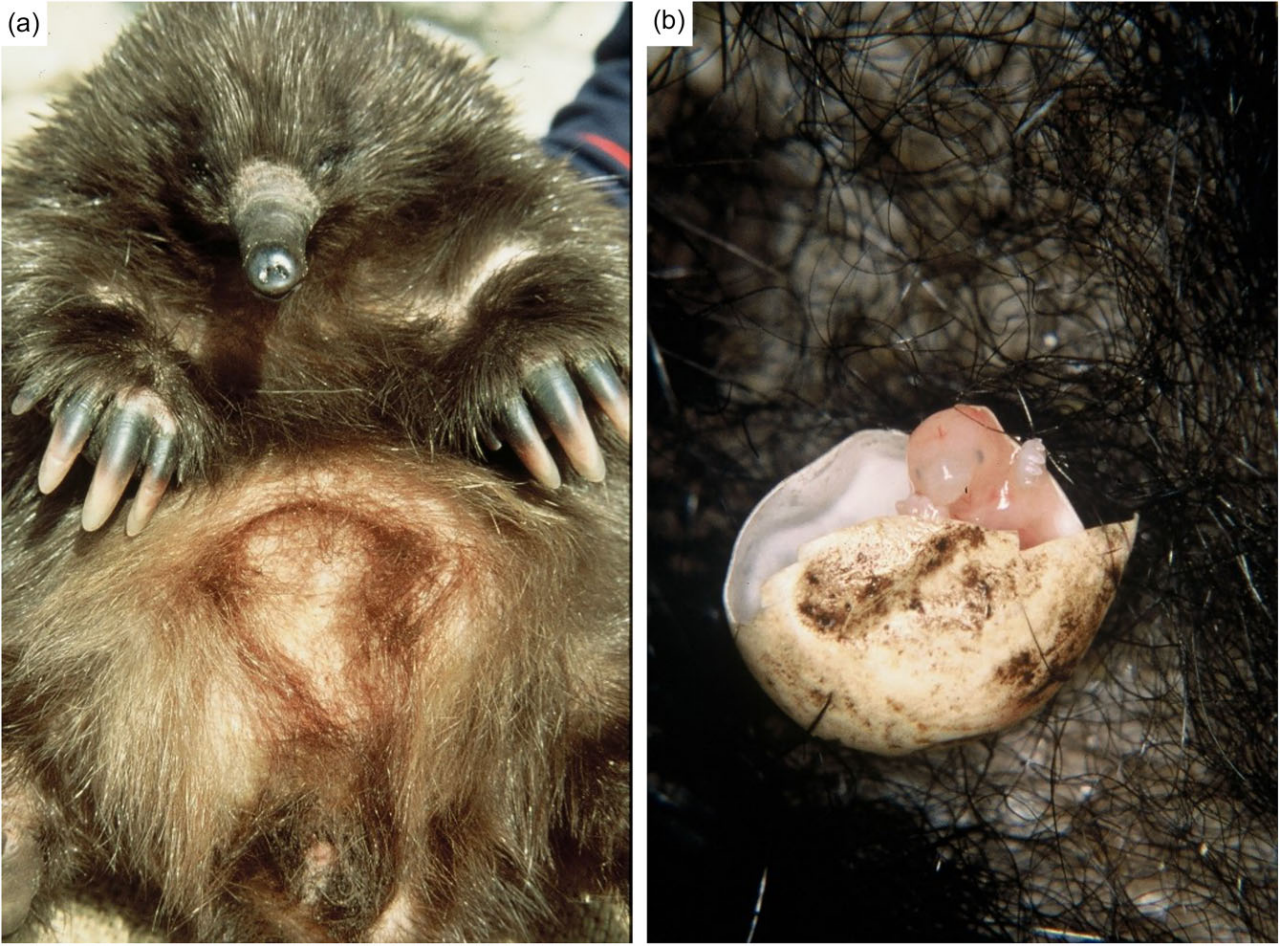
Photographs showing (a) an echidna pseudo-pouch and (b) an echidna hatchling. Image credit: Peggy Rismiller & Michael McKelvey.

Monotremes are the only egg-laying mammals and offer a unique perspective on reproduction and early development. The short-beaked echidna reproduces seasonally during a period lasting from June to September (Beard and Grigg 2000, Rismiller and McKelvey 2000, Nicol et al. 2005). The embryo undergoes a short gestation resulting in an altricial hatchling that lacks a functioning adaptive immune system (Griffiths 1978, Rismiller and Seymour 1991). The majority of early development occurs during a long lactation (∼160–210 days in echidnas) (Rismiller and McKelvey 2009) within the pseudo-pouch, a temporary pouch formed from the contraction of the abdominal muscles (Fig. 1). Monotremes lack nipples, instead nursing from the milk patch within the pseudo-pouch.

In monotremes and marsupials, the reproductive microbiome extends to the pouch—an environment critical for the development of their altricial young. Research into marsupial pouch microbiota has revealed compositional changes after the birth of the joey (Weiss et al. 2021). These changes are thought to be the result of the secretion of anti-microbial agents in the skin and milk, including cathelicidin, lysozyme, dermcidin, and immunoglobulins (Cheng and Belov 2017, Peel et al. 2017). This protective activity appears to modulate the pouch microbiota, potentially bringing about more favourable conditions for the young (Maidment and Eisenhofer 2023). While similar secretions have been identified in monotremes (Wang et al. 2011), reproductive microbiota have not yet been studied.

Information regarding the impact of captivity on animal microbiota is largely restricted to the gastrointestinal tract. For example, in short-beaked echidnas, southern hairy-nosed wombats, and Tasmanian devils, the gut microbiota of captive animals is less diverse than their wild counterparts (Cheng et al. 2015, Eisenhofer et al. 2021, Perry et al. 2022). However, recent studies have begun to investigate the relationship between captivity and reproductive microbiota. In black-footed ferrets, the taxonomic composition of reproductive microbiota was found to be significantly different between wild and captive animals and was correlated with reproductive success (Bornbusch et al. 2024). In captive koalas, neonatal mortality has been associated with overgrowths of specific pathogens within the pouch (Maidment et al. 2023), though it is unclear whether this phenomenon is unique to captive animals. Further research is needed to better understand the differences between the reproductive microbiota of captive and wild animals, as well as their potential effects on breeding success (Comizzoli et al. 2021).

The lack of a functioning adaptive immune system in monotreme hatchlings raises questions about pseudo-pouch microbiota during lactation. We hypothesized that secretions during breeding season or lactation would affect microbes in the pseudo-pouch, similar to what has been observed in the marsupial pouch. To investigate for the first time whether captivity, seasonal changes, or lactation affect the composition and diversity of the pseudo-pouch microbiota of short-beaked echidnas, wild and captive echidnas were swabbed during three different reproductive stages: outside of breeding season, within breeding season (June–September), and during lactation. This revealed extensive microbial changes within the pseudo-pouch after the onset of lactation.

## Materials and methods

### Sample collection

This study was carried out in accordance with requirements of the Taronga Animal Ethics Committee (AEC) for the ‘Collection of opportunistic samples for researchers from live animals during veterinary procedures or routine husbandry procedures’ (AEC 4a/02/18) and ‘Glucose levels and fibre fermentation in monotremes’ (AEC 3a/08/21). An overview of the major types of samples used in this study is provided in Table 1; see Supplementary files for full sample metadata. The majority of swabs were collected from captive echidnas at Taronga Zoo, NSW. Further swabs were taken from wild road-kill echidnas from Kangaroo Island (collected by Dr Peggy Rismiller) and the Adelaide Hills (sourced through EchidnaCSI). As the samples were opportunistically obtained from roadkill animals, the exact time of death is unknown; the animals had not yet undergone rigor mortis and were swabbed within a few hours post-mortem. Swabbing was undertaken using Copan FLOQSwabs® with gloved hands. Pouch swabs were taken from the pseudo-pouch in lactating animals and the corresponding abdominal region in non-lactating animals; oral swabs were taken from inside the beak; cloacal swabs were taken from the external cloacal opening and the internal cloaca; and environment samples were taken from dirt within captive echidnas’ enclosures, or from the ground next to deceased wild echidnas. Where swabs were collected within the lab, a lab negative control swab of the air was also taken for downstream identification of contaminants. Swabs were placed on ice before being frozen at −70°C to prevent DNA degradation.

**Table 1.** Overview of samples extracted within this study, sorted by sample type and species.

Sample type	Captive	Wild	Total
Pouch (lactating)	6	–	**6**
Pouch (inside of breeding season)	–	4	**4**
Pouch (outside of breeding season)	4	8	**12**
Oral	9	11	**20**
Cloacal	7	10	**17**
Environment	3	1	**4**
Total	**29**	**34**	**63**

### DNA extraction

All extractions took place in a still air class II biosafety cabinet that was decontaminated with 10% bleach. Total genomic DNA was extracted from swab samples using the ZymoBIOMICS^TM^ DNA Miniprep Kit according to the manufacturer's instructions, except that buffers were aliquoted prior to the introduction of samples into the hood in order to minimize contamination. In order to account for laboratory-based contamination, one extraction blank control in which no swab was added to the lysis tube was included with each set of extractions (total number of extraction blank controls = 10). Scissors used to cut swab tips into tubes were wiped on bleach-soaked paper towel in between each sample to avoid cross-contamination. Bead-beating step was carried out using a Disruptor Genie® cell disruptor for 15 min. DNA samples were stored at −20°C until required for further use.

### Amplicon library preparation and quantification

Library preparation was carried out according to the Earth Microbiome Project 16S Illumina Amplicon Protocol (Caporaso et al. 2012). The V4 region of the bacterial 16S rRNA gene was amplified in all samples using polymerase chain reaction (PCR). Additional negative (no DNA added; *n* = 10) and positive (DNA known to amplify successfully; from short-beaked echidna scat sample) controls were included in each set of PCR reactions. Each sample was amplified using the 515F forward primer: 5′-AATGATACGGCGACCACCGAGATCTACACTATGGTAATTGTGTGCCAGCMGCCGCGGTAA-3′, and the uniquely barcoded 806R reverse primer: 5′-CAAGCAGAAGACGGCATACGAGAT-nnnnnnnnnnnn-AGTCAGTCAGCCGGACTACHVGGGTWTCTAAT-3′, with the 12 *n*'s representing a unique barcode sequence for downstream identification (Caporaso et al. 2011).

Reactions containing 18.7 µl H_2_O, 2.5 µl 10X High Fidelity Buffer (Thermofisher), 1 µl 50 mM MgSO_4_, 0.2 µl 100 mM dNTP mix, 0.5 µl 10 µM forward primer, 0.1 µl Platinum™ Taq DNA Polymerase High Fidelity (Thermofisher), 1 µl sample DNA, and 1 µl 5 µM unique reverse primer were amplified using an initial denaturation step at 94°C for 3 min, followed by 35 cycles of denaturation at 94°C for 45 s, annealing at 50°C for 60 s, and elongation at 68°C for 90 s, with a final adenylation step at 68°C for 7 min. In order to assess whether the desired fragment of DNA (∼390 bp) had been successfully amplified, gel electrophoresis was performed using a 1.5% agarose gel with RedSafe™ Nucleic Acid Staining Solution (20 000×) (iNtRON Biotechnology) run at 100 V for 30 min.

For each sample (excluding PCR positive controls), 2 µl was sent to the Australian Cancer Research Facility (ACRF) for fluorometric quantification using a Qubit™. Samples were then pooled to equimolar concentration and cleaned using the Agencourt AMPure XP PCR purification system (Beckman Coulter) according to the manufacturer's instructions, before being sent to ACRF for a final quantification and quality assessment using an Agilent 2100 Bioanalyzer system. The samples were pooled to 4 nM and sequenced on an Illumina MiSeq (v2, 2 × 250 bp 15 M reads) at ACRF.

### Data analysis

Demultiplexed reads were quality-controlled and adaptor-trimmed using the FASTQ pre-processor programme fastp (Chen et al. 2018). The sequences were further processed and analysed using the QIIME2 (ver. 2024.10) bioinformatic pipeline (Bolyen et al. 2019). Paired-end reads were joined using VSEARCH (Rognes et al. 2016). Deblur (Amir et al. 2017) was used to denoise the reads into amplicon sequence variants (ASVs); a trim length of 252 bp was used based on visualization of the joined reads. The prevalence-based method in decontam (Davis et al. 2018) was used to identify putative contaminants (threshold score 0.5), which were removed from the feature table along with singletons (Figs S1 and S2). The taxonomic composition of negative control samples prior to contaminant removal is displayed in Fig. S3.

We created an alpha rarefaction plot in order to visualize the feature diversity within each sample up to a maximum depth of 35 000 (the median ASV frequency per sample). From this plot, a rarefaction depth of 6800 was selected for downstream diversity analysis. While this depth resulted in the loss of some diversity from the environment samples, little diversity was lost from other sample types and allowed for the retention of all pseudo-pouch samples. ASVs were taxonomically classified using the SILVA 16S V4 classifier (Quast et al. 2012) (ver. 138 515f 806r). Alpha diversity was estimated using Observed ASVs, Pielou's evenness, Shannon's diversity, and Faith's phylogenetic diversity (Faith 1992); beta diversity was estimated using unweighted and weighted UniFrac distances (Lozupone and Knight 2005). UniFrac distances were visualized using principal coordinate analysis (PCoA) plots. Significance of alpha and beta diversity was assessed using Kruskal–Wallis pairwise tests and Permutational Multivariate Analysis of Variance tests respectively. Statistical significance of differentially abundant taxa between groups was undertaken using the ANCOMBC R package (Mandal et al. 2015). Sourcetracker2 (Knights et al. 2011) (ver. 2.0.1) was used to estimate the contribution of oral, cloacal and environmental bacteria to the pouch microbiota. A rarefaction depth of 6800 was applied to all sinks. The per_sink_feature_assignments flag was used to assign QIIME2 feature IDs to each identified source ASV.

## Results

We performed 16S rRNA gene sequencing on a total of 63 samples: pseudo-pouch (*n* = 22), cloacal (*n* = 17), oral (*n* = 20), and habitat (*n* = 4). Swab samples were taken from wild and captive echidnas during three stages of the reproductive cycle (Table 1).

### Decontamination of the dataset

As DNA contamination can confound microbiota characterizations, and monotreme pseudo-pouch microbiota have not previously been studied, we first sought to validate the presence of microbes in the echidna pseudo-pouch. We used the decontam R package to identify putative contaminants within the dataset (Figs S1 and S2). This approach compares negative controls to biological samples in order to classify taxa as contaminants or non-contaminants. We captured contaminants introduced during the DNA extraction process using extraction blanks (i.e. samples to which no echidna swabs were added; *n* = 10) and during PCR using negative controls (i.e. PCR reactions to which no DNA was added; *n* = 10). Using a prevalence-based method with a threshold score of 0.5, 801 features were identified as contaminants out of a total of 12 328 features. These, along with ASVs identified as chloroplast or mitochondria, were removed from the dataset prior to further analyses.

### Short-beaked echidna pseudo-pouch microbiota is not significantly impacted by captivity or breeding season

**Figure 2.**
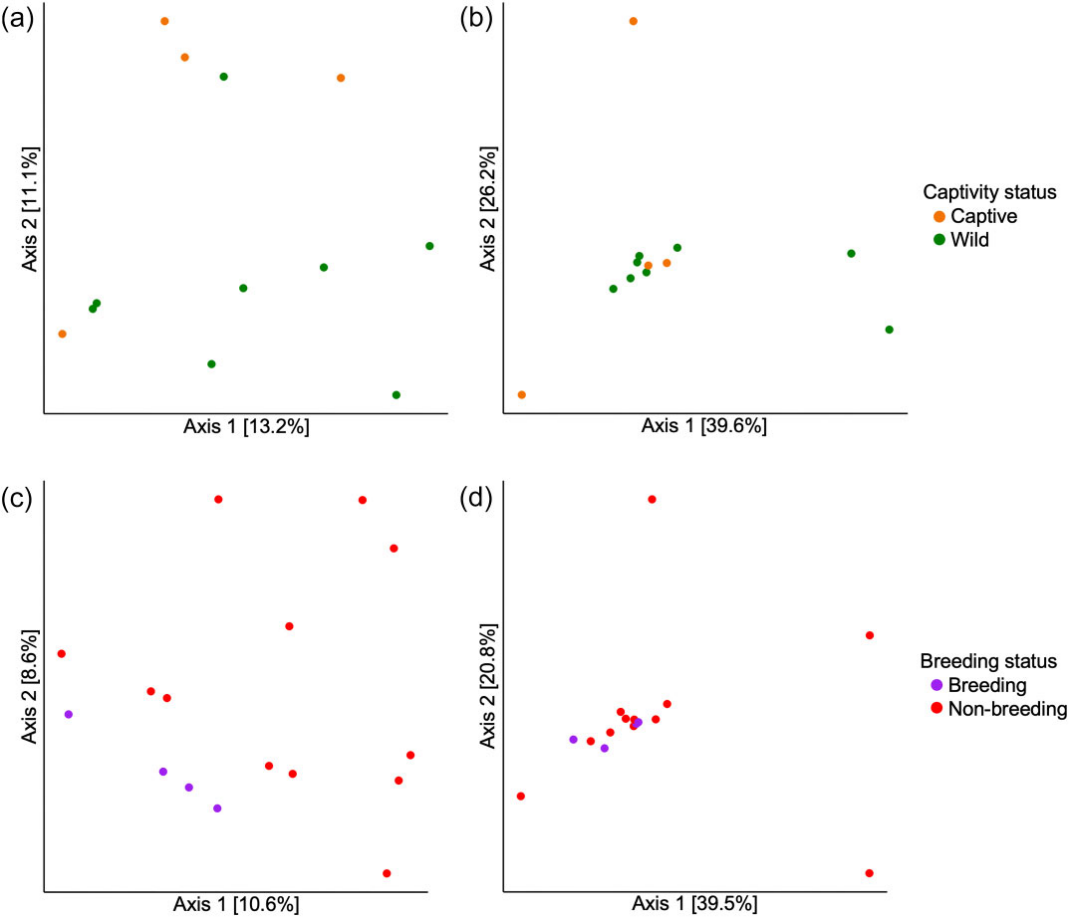
PCoA plots showing the composition of the pseudo-pouch microbiota according to captivity and breeding status. (a) Unweighted UniFrac distances between captive and wild echidnas sampled outside of breeding season or lactation. (b) Weighted UniFrac distances between captive and wild echidnas sampled outside of breeding season or lactation. (c) Unweighted UniFrac distances between wild echidnas inside vs outside of breeding season. (d) Weighted UniFrac distances between wild echidnas inside vs outside of breeding season.

Despite improvements in captive management and breeding, the survival rate of pouch young can be low. To explore whether this may be associated with altered microbiota in captive animals, we compared the microbial diversity in the pseudo-pouch between wild and captive echidnas. Microbial diversity was analysed using four different alpha diversity metrics: species richness (i.e. the number of species, measured using observed ASVs and Shannon's index), species evenness (i.e. the distribution of species abundances, measured using Pielou's evenness), and phylogenetic diversity (a measure of diversity that incorporates branch length, measured using Faith's phylogenetic diversity). Samples collected during breeding season (*n* = 4) or lactation (*n* = 6) were excluded so as not to skew the results, leaving four captive and eight wild samples. Surprisingly, no significant differences were found between samples derived from wild and captive pseudo-pouches for any of our tested metrics of alpha diversity (Faith's phylogenetic diversity, richness, evenness) (Fig. S4; *P* > .05). Similarly, beta diversity was not significantly different between the groups (Fig. 2; unweighted UniFrac distance *P* = .199, weighted UniFrac distance *P* = .808).

Next, we considered whether hormonal changes during breeding season affect the microbiota in the pseudo-pouch. However, the microbiota of wild echidnas within (*n* = 4) and outside (*n* = 12) of breeding season showed no statistically significant differences for any measure of alpha diversity (Fig. S5; *P* > .05) or beta diversity (Fig. 2; unweighted UniFrac distance *P* = .189, weighted UniFrac distance *P* = .585). Overall, our evidence suggests that neither breeding season nor captivity have a significant impact on the microbial diversity or composition of echidna pseudo-pouch microbiota, though these results may be affected by low sample sizes (*n* = 12 for captive/wild, *n* = 16 for breeding/non-breeding).

### Lactation-associated changes in the pseudo-pouch microbiota of wild and captive short-beaked echidnas

**Figure 3.**
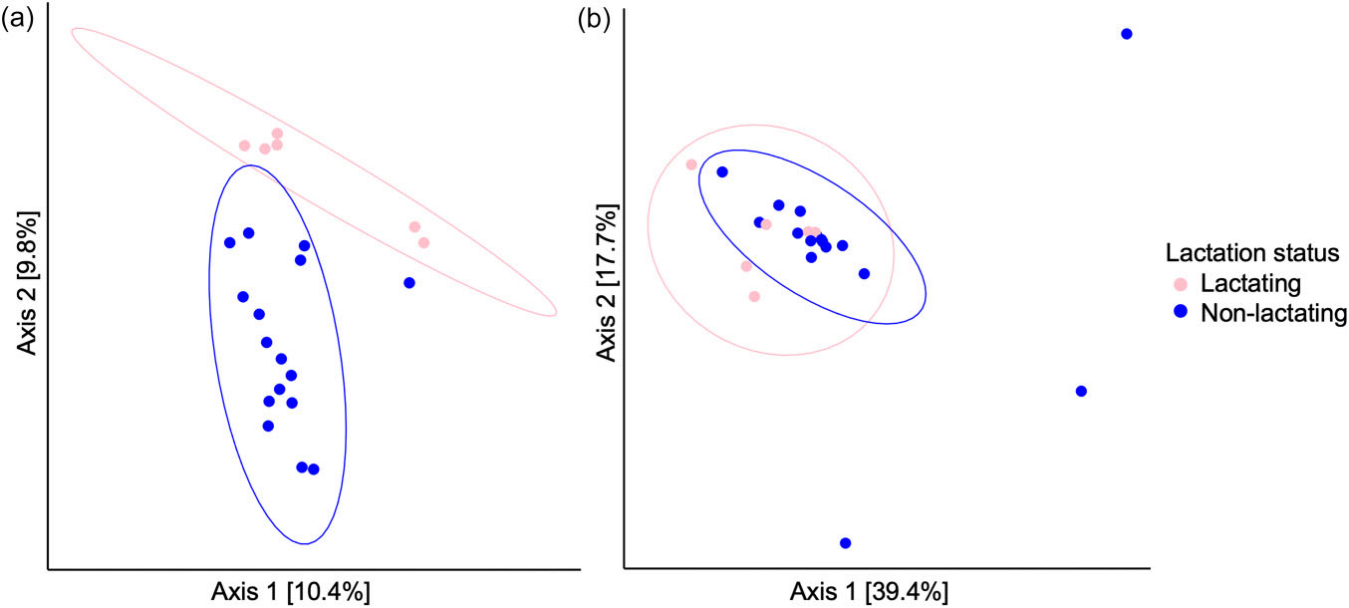
PCoA plots showing the composition of the pseudo-pouch microbiota in lactating and non-lactating echidnas. (a) Unweighted UniFrac distances showing lactating vs non-lactating clusters. (b) Weighted UniFrac distances between lactating and non-lactating echidnas.

We compared the diversity and taxonomic composition of pseudo-pouch microbiota samples from lactating and non-lactating (including breeding and non-breeding) echidnas. Although measures of alpha diversity showed no significant differences (Fig. S6), analysis of beta diversity revealed that lactating pseudo-pouch samples were broadly dissimilar from their non-lactating counterparts. PCoA using unweighted UniFrac distances showed distinct clustering of lactating and non-lactating samples (Fig. 3a). This clustering was less distinct with weighted UniFrac distances (Fig. 3b). For both metrics, the microbial composition of the pseudo-pouch was found to be significantly different between lactating and non-lactating samples (unweighted UniFrac *P* = .006; weighted UniFrac *P* = .028).

**Figure 4.**
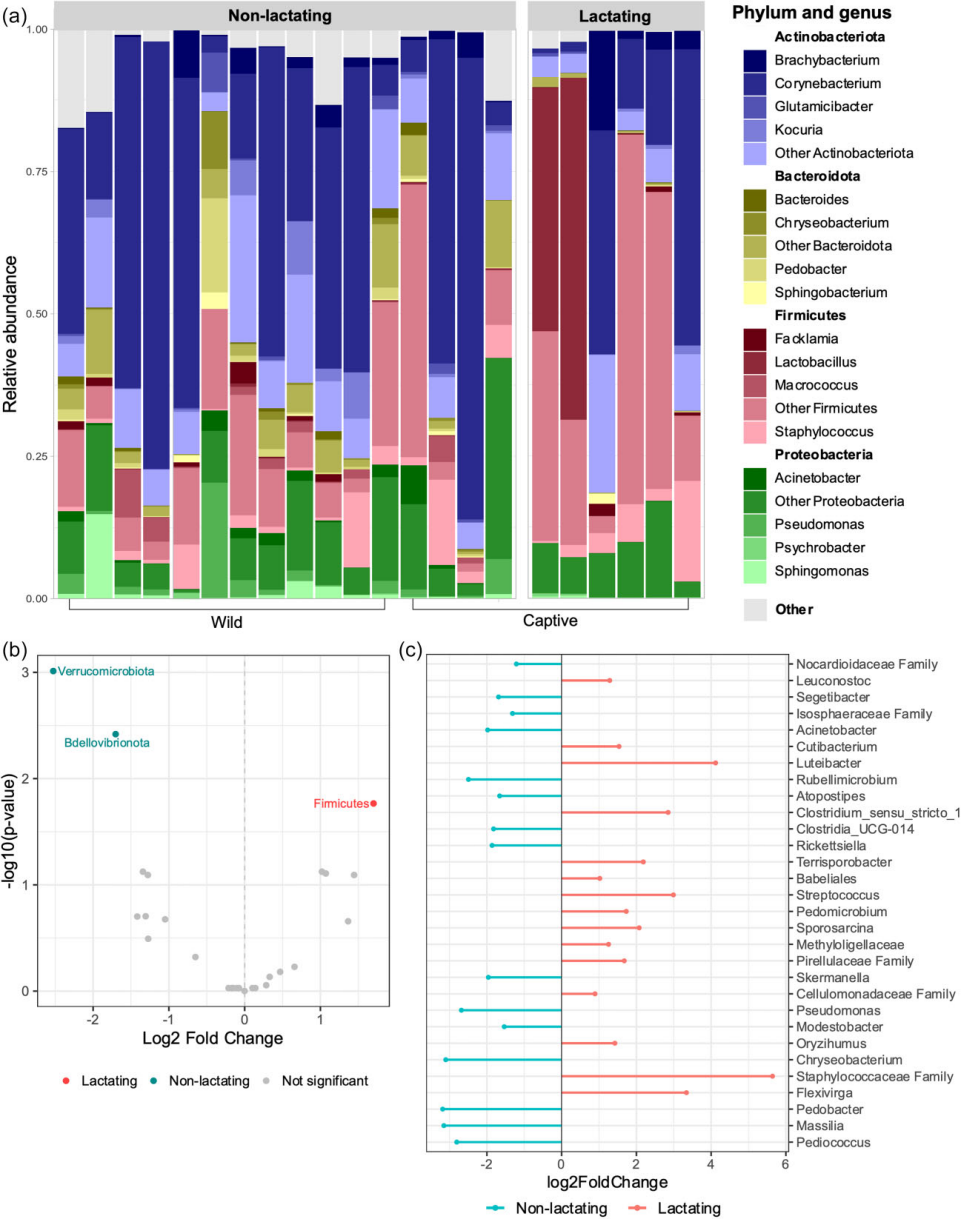
Taxonomy of the short-beaked echidna pseudo-pouch microbiome comparing bacterial composition in non-lactating vs lactating animals. (a) Relative proportions of bacteria displayed at the genus level, grouped according to phylum. (b) Volcano plot showing phyla identified as significantly differentially abundant by ANCOMBC. (c) Lollipop plot showing the top 30 most prevalent genera identified as significantly differentially abundant by ANCOMBC.

To better understand the compositional changes observed during lactation, we investigated the taxonomic composition of the different groups (Fig. 4). Although all pseudo-pouch groups were dominated by taxa from the same three phyla—Actinobacteria, Proteobacteria, and Firmicutes—non-lactating samples primarily contained Actinobacteria (52.3%) and relatively little Firmicutes (17.9%), whereas in the lactating pseudo-pouch, Firmicutes was the most prevalent phylum (54.9%) and Actinobacteria constituted only 33.8% of the microbiota (Fig. 4a). The increase of Firmicutes taxa in the lactating group was found to be statistically significant (Fig. 4b; ANCOMBC *q* = 0.017). Other significant changes included decreases in Bdellovibrionota (*q* = 0.004) and Verrucomicrobiota (*q* = 0.001), though these phyla made up <1% of bacteria in both groups. Overall, the lactating pseudo-pouch appeared to be less taxonomically diverse with only three phyla constituting 98% of observed bacteria; however, no significant differences in phylogenetic diversity were detected between lactating and non-lactating pseudo-pouch samples (Fig S6, Faith's phylogenetic diversity *P* = .065).

In order to gain a more detailed understanding of the differences between lactating and non-lactating pseudo-pouch microbiota, the samples were further analysed at the genus level (Fig. 4a). We observed substantial variation in diversity between individuals. Of the 22 pseudo-pouch samples, 10 were occupied predominantly (i.e. >50%) by a single bacterial genus (typically *Corynebacterium* spp.). A further seven samples had relatively even levels of a small number of bacterial taxa. The remaining five samples were highly diverse with no apparent dominant taxa. Non-lactating pseudo-pouch samples were predominantly populated by *Corynebacterium* (46.9% in breeding, 36.5% in non-breeding), though some samples were highly diverse with no clear dominant species. The second most prevalent genus within breeding season was *Tychonema* (5.0%) whereas outside of breeding season it was *Staphylococcus* (4.3%). The taxonomic composition of the lactating pseudo-pouch microbiota was substantially altered. The proportion of *Corynebacterium* trended downward (37.0% in non-lactating vs 20.4% in lactating), whereas a number of other taxa trended upward, including *Staphylococcaceae* (0.21% in non-lactating vs 20.64% in lactating), *Enterobacteriaceae* (0.69% in non-lactating vs 6.08% in lactating), and *Lactobacillus* (0.13% in non-lactating vs 5.22% in lactating), though none of these observations were statistically supported (*q* > 0.05). A total of 48 genera were found to be significantly differentially abundant in lactating animals as compared to non-lactating animals. Significant changes were generally among low-prevalence genera. Among the changes were an increase in Enterococcus (0.40% in non-lactating vs 0.76% in lactating; *q* = 0.006), Streptococcus (0.05% in non-lactating vs 0.49 in lactating; *q* = 0.012) and Luteibacter (0.14% in non-lactating vs 0.39% in lactating; *q* = 8.236e−8), and a reduction in Pseudomonas (2.68% in non-lactating vs 0.06% in lactating; *q* = 1.092e−7), Macrococcus (1.60% in non-lactating vs 0% in lactating; *q* = 3.812e−9), and Acinetobacter (1.41% in non-lactating vs 0.06% in lactating; *q* = 0.033) (Fig. 4c). Of the 48 differentially abundant genera, the majority (66.6%) represented reductions in relative abundance in lactating animals.

### The pseudo-pouch microbiota is a distinct microbial niche

Next, we tested the extent to which other bodily niches and/or the animal's habitat contributed to changes in the pseudo-pouch microbiota. This included the cloaca, due to the egg passing through this area before being laid into the pseudo-pouch; the mouth, due to the licking behaviour of the dam towards the puggle; dirt from echidna sampling locations (environment), due to the potential exposure of the pseudo-pouch and the milk patch to the ground; and extraction and PCR negative controls, to ensure that any microbes introduced from the laboratory environment were not assumed to be sourced from the animal or its habitat.

**Figure 5.**
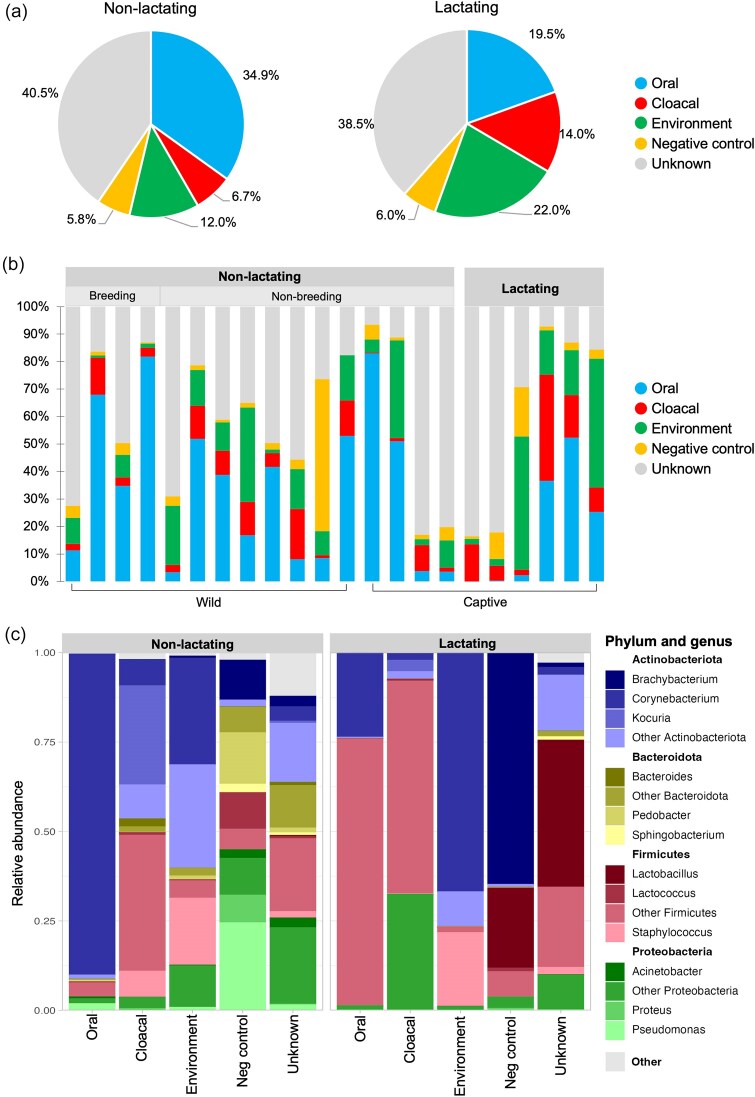
Investigating the origin of the pseudo-pouch microbiome (Sourcetracker2). (a) Pie chart showing the average contribution (%) of bacteria from the cloaca, mouth, environment, and negative controls to the pseudo-pouch microbiome in non-lactating and lactating animals. (b) Bar graph showing the relative contribution of sources in each pseudo-pouch sample, grouped according to lactation, breeding, and captivity status. (c) Taxonomic composition of bacteria contributed by each source group in lactating and non-lactating echidnas.

In order to see if any one source group was more similar to the pseudo-pouch than another, we performed beta diversity analysis. This showed that all source groups were significantly different from the pseudo-pouch regardless of lactation status (Fig. S7; *P* < .05). The only exception to this was that the non-lactating pouch was not found to be significantly different from environmental samples when using weighted UniFrac distances (*P* = .066); however, significant differences were seen when using unweighted UniFrac distances (*P* = .036). We then used Sourcetracker2, a programme designed to evaluate the occurrence of microbial seeding between different niches (Fig. 5). Taxa derived from the mouth decreased from 34.9% in non-lactating samples to 19.5% in lactating samples (Fig. 5a). Conversely, taxa derived from the cloaca showed a modest increase (7.3%) during lactation. For both groups, pseudo-pouch taxa were found to be derived primarily from unknown sources. This suggests that the echidna pseudo-pouch, particularly during lactation, is a specialized microbial niche. The proportion of taxa derived from negative controls (i.e. lab contamination) was relatively small for each group. The contribution of each source varied considerably on a per-sample basis (Fig. 5b).

We then examined the taxonomic composition of the bacteria contributed by each source group (Fig. 5c). The proportion of oral-derived taxa comprised by Actinobacteria appeared to decrease between non-lactating (82.09%) and lactating (33.83%) animals, though this change was not significant (*q* = 0.073). Modest but significant changes were observed for Proteobacteria (9.75% in non-lactating vs 9.83% in lactating; *q* = 0.043) and Bacteroidota (1.68% in non-lactating vs 2.74% in lactating; *q* = 0.004e−02). For taxa sourced from the cloaca, non-lactating samples were evenly split between Actinobacteria (42.09%; primarily *Kocuria* spp.) and Firmicutes (47.85%) with relatively little Proteobacteria (4.23%). Conversely, in lactating samples, the proportion of Proteobacteria (37.47%) was significantly increased (*q* = 0.010) while the proportion of Actinobacteria trended downwards (non-lactating 42.09% vs lactating 10.50%), though this decrease was not significant (*q* = 0.594). The composition of environment-derived bacteria in lactating and non-lactating echidnas exhibited no significant differences at the phylum level. Bacteria derived from unknown sources showed a higher proportion of Firmicutes (particularly *Lactobacillus* spp.) in lactating echidnas (*q* = 0.022).

## Discussion

The unique biology of monotremes provides invaluable information about the role of lactation in shaping the microbial environment of young lacking functional adaptive immunity (Comizzoli et al. 2021). In this study we investigated for the first time how the microbiota of the echidna pseudo-pouch changes during the breeding cycle and lactation, and whether captivity influences the pseudo-pouch microbiota.

In comparison to non-lactating animals, the pseudo-pouches of lactating echidnas exhibited significant changes in taxonomic composition and beta diversity. This suggests that only certain bacterial taxa were permitted to remain in the pseudo-pouch during the critical lactation period. For example, during lactation, the proportion of Firmicutes was significantly increased at the expense of Actinobacteria. This contrasts with previous studies of marsupial pouches during lactation, including the quokka, tammar wallaby and southern hairy-nosed wombat, where Actinobacteria made up the vast majority (>80%) of all microbial diversity (Charlick et al. 1981, Chhour et al. 2010, Weiss et al. 2021). This difference may be due to monotreme oviparity and the passage of the egg through the cloaca. Our analysis suggests that the interaction between the cloaca and pseudo-pouch microbiota is altered during lactation, both in terms of the proportion and taxonomy of bacteria. The proportion of pseudo-pouch bacteria sourced from the cloaca was greater during the lactation period than in non-lactating animals, potentially due to the egg transferring cloacal microbes into the pseudo-pouch. Furthermore, during lactation, most taxa sourced from the cloaca were Firmicutes spp., whereas in non-lactation there was a relatively even split between Firmicutes and Actinobacteria spp. It is possible that the broad increase in Firmicutes and reduction in Actinobacteria seen in the lactating pseudo-pouch are the result of exchange with cloacal microbiota after the egg is laid.

Further changes were observed at the genus level. Significant differences were most commonly observed for low-prevalence taxa. Many of the genera found to be significantly reduced during lactation were associated with soil, such as Flavobacterium, Pedobacter, and Acinetobacter. A number of significantly increased taxa were lactic acid bacteria and/or are known commensals of the lower reproductive tract in humans, such as Peptostreptococcus, Streptococcus, and Enterococcus (Koedooder et al. 2019). Lactic acid bacteria are considered probiotic as they often provide protection by outcompeting pathogens and provide a healthy foundation for the infant gastrointestinal microbiome (Cheng and Belov 2017, Jin Song et al. 2019, Wang et al. 2021). The proliferation of such commensal species, in addition to the reduction in soil microbes, may represent a transition into a more hospitable setting for pouch young development (Peel et al. 2016). Surprisingly, the largest changes in relative abundance, such as substantial reductions in *Corynebacterium* and increases in *Lactobacillus*, were not found to be statistically significant. Lactation-associated reductions in *Corynebacterium* spp. have been observed in the southern hairy-nosed wombat (Weiss et al. 2021), though the study did not perform differential abundance analysis so it is unclear whether the reported changes were statistically significant. This genus contains a range of species from human skin commensals to the pathogenic *C. diphtheriae*. In our study, *Corynebacterium* species could only be identified to the genus level, and it therefore remains unknown whether taxa that showed a reduced abundance were beneficial or harmful. The lack of significance in our study may be related to sample size; many of the most dramatic changes were observed in only two individuals.

The shift that we observed between lactating and non-lactating echidna pseudo-pouches is consistent with research in marsupial species. Tammar wallabies undergo broad changes in their pouch microbiota composition leading up to and after birth, which involve reductions in overall bacterial richness and Gram-negative species in particular (Old and Deane 1998, Chhour et al. 2010). A similar phenomenon has been observed in the Tasmanian Devil, where the pouch exhibits reduced abundances of potential pathogens during lactation (Peel et al. 2016). One proposed mechanism for these changes is skin secretions. These secretions contain antimicrobial peptides including lysozyme, dermcidin, and cathelicidins, the levels of which have been shown to change during different reproductive stages (Cheng and Belov 2017). Lysozyme has been shown to bring about decreases in Firmicutes and increases in Bacteroides species in the humans gastrointestinal tract (Maga et al. 2012)—though this is the opposite of what we observed in the lactating echidna pseudo-pouch. Cathelicidins may work to specifically target pathogens while not affecting commensals, thus bringing about a beneficial microbial environment for pouch young development (Peel et al. 2016). Cathelicidins have been found in the platypus (Wang et al. 2011) and several ‘cathelicidin-like’ genes have been annotated in the echidna genome, raising the possibility that these peptides are key to shaping pseudo-pouch microbiota during lactation. Another potential mechanism of pouch microbiota alteration is that of the milk itself. Marsupial and monotreme milk contains a range of antimicrobial agents including immunoglobulin, lysozyme, transferrin, and host defence peptides (Stannard et al. 2020). In marsupials, these antimicrobials are established to protect the infant from infection after ingestion, but may also promote a beneficial microbial environment within the pouch (Cheng and Belov 2017). Echidna milk also contains the monotreme-specific peptides MLP and EchAMP, which have demonstrated species-specific activity against a number of both Gram-positive and Gram-negative bacteria (Bisana et al. 2013, Enjapoori et al. 2014). Currently, we do not understand how these antimicrobial compounds impact monotreme pseudo-pouch microbiota.

Surprisingly, our study did not detect any significant differences in the diversity nor the composition of the pseudo-pouch microbiota between captive and wild individuals. Considering the small sample size in this study, further research is needed to understand the effects of captivity on the echidna pouch microbiota. There is very little research into the impacts of captivity on pouch microbiota in any species—however, our result contrasts with what has been observed in the Tasmanian devil pouch (Cheng et al. 2015) as well as the gastrointestinal tract of several mammals including echidnas, where captivity brought about decreased diversity and major microbial changes (McKenzie et al. 2017, Perry et al. 2022). The importance of reproductive microbiota in infant immunity is well documented in humans (Mueller et al. 2015, Nyangahu and Jaspan 2019) but has not been investigated in marsupials or monotremes. Captive-bred puggles face several health challenges not found in wild echidnas, including high coccidia burden leading to secondary yeast infections (Tobias 2019). Our observation that there were no significant differences between the pseudo-pouch microbiota of wild and captive echidnas suggests that other factors contribute to these health issues.

We considered whether the microbiota of other body parts could contribute to the echidna pseudo-pouch microbiota. In particular, we expected that cloacal microbes would be transferred into the pseudo-pouch during oviposition. Oral microbes (derived from pouch-licking behaviour) and microbes from an animal's environment would also be expected to contribute to the pseudo-pouch microbiota. However, our results show that the pseudo-pouch is highly distinct; ∼40% of pouch of microbes could not be assigned a specific source regardless of lactation status. This is similar to what has been observed in marsupials; when comparing marsupial milk/pouch samples to other body regions, the beta diversity of reproductive microbiota was significantly different to that of other body sites e.g. the mouth or cloaca (Cheng et al. 2015, Weiss et al. 2021). Several of the pseudo-pouch microbes found to have been sourced from the environment have been characterized as soil bacteria, such as *Brevibacterium, Glutamicibacter*, and *Streptomyces* (Forquin-Gomez et al. 2014, Viaene et al. 2016, Andrey et al. 2018). Surprisingly, the proportion of environment-derived bacteria increased modestly in lactating animals whilst remaining largely taxonomically unchanged. This suggests that many environmental bacteria are not impacted by the broad changes affecting the pseudo-pouch microbiota in lactation. Our hypothesis that the licking behaviour of echidna dams would increase the proportion of oral-derived microbes in the pseudo-pouch during lactation was not supported; in fact, the oral contribution was decreased during lactation. This contrasts with theories of marsupial development that posit that licking plays a significant role in creating an altered pouch environment for the neonate (Ambatipudi et al. 2008, Weiss et al. 2021). Despite this decrease, oral microbes still made up a large proportion of the pseudo-pouch microbiota, particularly in non-lactating animals. As previously discussed, the cloacal contribution to the pseudo-pouch microbiota was increased during lactation. This result is consistent with what has been observed in Tasmanian devils, where lactating pouches contained more gut-derived bacteria than non-lactating pouches, presumably due to faecal contamination from the pouch young (Peel et al. 2016). This suggests that contamination from the digestive tract in the pseudo-pouch is not a cause for concern regarding neonatal development. One potential source not considered due to a lack of sampling access was the neonate itself. Future work should investigate the extent to which the puggle microbiota shapes the pseudo-pouch and vice versa.

The data presented in this study were generated solely using 16S rRNA amplicon sequencing. While this approach is widely used for microbiota research, it has several well-documented limitations. PCR amplification and sequencing both introduce bias towards particular bacterial species; no 16S primer pair is truly universal, and next-generation sequencing platforms respond differently to sequence attributes such as GC content (Salipante et al. 2014, Tremblay et al. 2015). Furthermore, 16S amplicon sequencing is only able to evaluate the relative abundances of bacterial taxa rather than absolute quantities, thus providing an incomplete understanding of community dynamics. Another major limitation of this approach is a lack of functional information. While bioinformatics tools have been developed to infer functional profiles from taxonomic composition (Aßhauer et al. 2015, Douglas et al. 2020), concerns have been raised regarding their accuracy, particularly for non-human animal hosts (Sun et al. 2020, Matchado et al. 2024). Future research should incorporate quantitative and functional approaches, e.g. quantitative PCR and shotgun metagenomics, in order to fully understand the impact of lactation on the echidna pseudo-pouch microbiota.

## Conclusion

Changes in pouch microbiota associated with lactation have been reported in various mammals and are important for the development and protection of neonates. Monotremes are the only egg-laying mammals but share with marsupials an extended lactation period that commences at a much earlier stage in development. This first characterization of the short-beaked echidna pseudo-pouch microbiota has revealed changes in taxonomic composition during lactation, similar to what has been observed in marsupials. Surprisingly, neither breeding season nor captivity resulted in changes to pseudo-pouch microbiota. Overall, our results suggest that the pseudo-pouch microbiota form a distinct community shaped by lactation and external microbial sources. Importantly, we did not detect any significant differences between captive and wild echidna pseudo-pouch microbiota, suggesting that this is unlikely to contribute to captivity-related health issues in infant echidnas. A better understanding of the molecular mechanisms by which lactation alters pseudo-pouch microbiota will contribute important insights into early echidna development and provide potential candidates for biomedical translation.

## Supplementary Material

fiaf036_Supplemental_Files

## Data Availability

The datasets presented in this study can be found at PRJNA1073668. All QIIME2 and R code used for analyses and figure generation are available on GitHub: https://github.com/isa-wilson/echidna_pouch_microbiome.
