# Supplementary material for: Microbiota changes in lactation in the short-beaked echidna (Tachyglossus aculeatus)
Source: FEMS Microbiol Ecol. 2025 Apr 7;101(5):fiaf036. doi: 10.1093/femsec/fiaf036 (PMC12001884; doi:10.1093/femsec/fiaf036)
Supplement: fiaf036_Supplemental_Files [file fiaf036_supplemental_files.zip › FEMS_SI.docx]

Fig. S1. Histogram of decontam scores.

**Fig. S2.** Prevalence/prevalence plot of taxa identified in biological samples vs negative controls. Taxa identified by decontam as contaminants (score >0.5) are shown in blue.

Fig. S3. Taxonomic composition of negative controls prior to contaminant removal. Taxa are displayed to the genus level. Top 15 most prevalent taxa are listed to the right of the plot.

Fig. S4. Alpha diversity analysis of captive and wild pseudo-pouch samples. Analysis was restricted to non-lactating and non-breeding season samples only. a) Species richness measured by observed OTUs; b) species richness measured by Shannon’s index; c) species evenness measured by Pielou’s evenness; d) phylogenetic diversity measured by Faith’s PD.

Fig. S5. Alpha diversity analysis of pseudo-pouch samples taken within vs outside of breeding season. Analysis was restricted to wild samples only. a) Species richness measured by observed OTUs; b) species richness measured by Shannon’s index; c) species evenness measured by Pielou’s evenness; d) phylogenetic diversity measured by Faith’s PD.

Fig. S6. Alpha diversity analysis of lactating vs non-lactating pseudo-pouch samples. a) Species richness measured by observed OTUs; b) species richness measured by Shannon’s index; c) species evenness measured by Pielou’s evenness; d) phylogenetic diversity measured by Faith’s PD.

Fig. S7. PCoA showing the compositional similarity between the pseudo-pouch microbiome and three potential sources of its microbial diversity (cloaca, mouth, environment) plus negative controls using a) unweighted UniFrac.distances and b) weighted UniFrac distances.

**Fig. S8.** Heatmap showing per-sample proportion of pseudo-pouch microbiota from five potential sources: cloaca, mouth, environment, and negative controls (Sourcetracker2). Sinks are labelled according to captivity and breeding/lactation status.

**Table S1.** Statistical significance of alpha diversity analyses on captive vs wild non-lactating pseudo-pouches sampled outside of breeding season or lactation.

| Alpha diversity metric | H | p-value | q-value |
| --- | --- | --- | --- |
| Observed OTUs | 0.029 | 0.865 | 0.865 |
| Shannon Diversity | 0.0 | 1.0 | 1.0 |
| Pielou’s Evenness | 0.0 | 1.0 | 1.0 |
| Faith’s Phylogenetic Diversity | 0.260 | 0.610 | 0.610 |

**Table S2.** Statistical significance of beta diversity (unweighted and weighted UniFrac) on captive and wild non-lactating pseudo-pouches sampled outside of breeding season or lactation.

| Test | Sample Size | Permutations | pseudo-F | p-value | q-value |
| --- | --- | --- | --- | --- | --- |
| **Unweighted UniFrac** | 12 | 999 | 1.128 | 0.199 | 0.199 |
| **Weighted UniFrac** | 12 | 999 | 0.393 | 0.808 | 0.808 |

**Table S3.** Statistical significance of alpha diversity analyses on wild echidna pseudo-pouch samples inside vs outside of breeding season.

| Alpha diversity metric | H | p-value | q-value |
| --- | --- | --- | --- |
| Observed OTUs | 1.470 | 0.225 | 0.225 |
| Shannon Diversity | 0.368 | 0.544 | 0.544 |
| Pielou’s Evenness | 0.941 | 0.332 | 0.332 |
| Faith’s Phylogenetic Diversity | 1.779 | 0.182 | 0.182 |

**Table S4.** Statistical significance of beta diversity (unweighted and weighted UniFrac) on wild echidna pseudo-pouch samples inside vs outside of breeding season.

| Test | Sample Size | Permutations | pseudo-F | p-value | q-value |
| --- | --- | --- | --- | --- | --- |
| **Unweighted UniFrac** | 16 | 999 | 1.119 | 0.189 | 0.189 |
| **Weighted UniFrac** | 16 | 999 | 0.622 | 0.585 | 0.585 |

**Table S5.** Statistical significance of alpha diversity analyses on lactating and non-lactating pseudo-pouch samples.

| Alpha diversity metric | H | p-value | q-value |
| --- | --- | --- | --- |
| Observed OTUs | 2.397 | 0.122 | 0.122 |
| Shannon Diversity | 1.571 | 0.210 | 0.210 |
| Pielou’s Evenness | 0.658 | 0.417 | 0.417 |
| Faith’s Phylogenetic Diversity | 3.397 | 0.065 | 0.065 |

**Table S6.** Statistical significance of beta diversity (unweighted and weighted UniFrac) on lactating vs non-lactating pseudo-pouch samples.

| Test | Sample Size | Permutations | pseudo-F | p-value | q-value |
| --- | --- | --- | --- | --- | --- |
| **Unweighted UniFrac** | 22 | 999 | 1.861 | 0.006 | 0.006 |
| **Weighted UniFrac** | 22 | 999 | 3.037 | 0.028 | 0.028 |

**Table S7.** Statistical significance of beta diversity analysis (unweighted UniFrac) on pouch microbiome samples and their proposed sources (cloaca, mouth, environment, negative controls).

| Group 1 | Group 2 | Sample Size | Permutations | pseudo-F | p-value | q-value |
| --- | --- | --- | --- | --- | --- | --- |
| Non-lactating pseudo-pouch | Cloacal | 33 | 999 | 5.476 | 0.001 | 0.002 |
|  | Oral | 36 | 999 | 3.730 | 0.001 | 0.002 |
|  | Environment | 20 | 999 | 1.399 | 0.036 | 0.036 |
|  | Neg controls | 37 | 999 | 4.101 | 0.001 | 0.002 |
| Lactating pseudo-pouch | Cloacal | 23 | 999 | 3.495 | 0.001 | 0.002 |
|  | Oral | 26 | 999 | 2.356 | 0.008 | 0.009 |
|  | Environment | 10 | 999 | 1.906 | 0.029 | 0.031 |
|  | Neg controls | 27 | 999 | 2.477 | 0.001 | 0.002 |

**Table S8.** Statistical significance of beta diversity analysis (weighted UniFrac) on pouch microbiome samples and their proposed sources (cloaca, mouth, environment, negative controls).

| Group 1 | Group 2 | Sample Size | Permutations | pseudo-F | p-value | q-value |
| --- | --- | --- | --- | --- | --- | --- |
| Non-lactating pseudo-pouch | Cloacal | 33 | 999 | 16.348 | 0.001 | 0.002 |
|  | Oral | 36 | 999 | 30.027 | 0.001 | 0.002 |
|  | Environment | 20 | 999 | 2.538 | 0.066 | 0.066 |
|  | Neg controls | 37 | 999 | 17.157 | 0.001 | 0.002 |
| Lactating pseudo-pouch | Cloacal | 23 | 999 | 4.692 | 0.003 | 0.004 |
|  | Oral | 26 | 999 | 11.643 | 0.001 | 0.002 |
|  | Environment | 10 | 999 | 3.876 | 0.008 | 0.009 |
|  | Neg controls | 27 | 999 | 8.324 | 0.001 | 0.002 |
